# Validating *MARK2 *Gene Polymorphism as a Predictor of Response to Lithium Treatment in Bipolar Patients

**DOI:** 10.52547/ibj.26.2.110

**Published:** 2021-12-26

**Authors:** Sara Sadat Aghabozorg Afjeh, Jamal Shams, Safar Hamednia, Behzad Boshehri, Asmaolhosna Amini, Amin Omrani, Mir Davood Omrani

**Affiliations:** 1 Department of Medical Genetics, Shahid Beheshti University of Medical Sciences, Tehran, Iran;; 2 Behavioral Research Center, Shahid Beheshti University of Medical Sciences, Tehran, Iran;; 3 Department of Psychiatry, Urmia University of Medical Sciences, Urmia, Iran;; 4 Department of Forensic Medicine and Toxicology, Urmia University of Medical Sciences, Urmia, Iran;; 5 Sara Medical Genetics Laboratory, Tehran, Iran;; 6 Faculty of Medicine of Tehran University of Medical Sciences, Tehran, Iran;; 7 Urogenital Stem Cell Research Center, Shahid Beheshti University of Medical Sciences, Tehran, Iran

**Keywords:** Biomarkers, Bipolar, Lithium, Genotyping

## Abstract

**Background:**

Lithium is a therapeutic option for the treatment of the acute phase of the bipolar disorder and long-term management of this disorder. However, it is estimated that 10 to 60% of patients do not properly response to this medication.

**Methods:**

To investigate the role of *MARK2 *gene in response to lithium, we genotyped the *MARK2 *rs10792421 polymorphism in Iranian bipolar patients using ARMS-PCR.

**Results:**

Results of this study showed a significant association of this polymorphism with response to lithium. The A allele was more frequent in the responder than the non-responder group and also in the semi- responder group compared to the non-responder group in the codominant model of analysis. AA and AG genotypes were more frequent in both the responder and semi-responder groups compared to the non-responder group in dominant model of analysis.

**Conclusion:**

Based on the findings of the current study, the rs10792421 variant of *MARK2 *gene could be considered as a potential biomarker for predicting the treatment outcome of bipolar disorder type 1 in Iranian population.

## INTRODUCTION

Lithium, as a well-established therapeutic option, has long been used for the management of severe mental illnesses^[^^1^^]^. Regarding mood-stabilizing properties, lithium has been known as the main medical option for the acute and long-term treatment of bipolar disorder^[^^2^^]^. However, it is estimated that 10 to 60% of patients do not show an appropriate response to the medication, and the introduction of new medications has not improved these numbers^[^^3^^]^. Nonadherence of patients to lithium confers a high risk of relapse and may make them susceptible to rebound depression and mania^[^^4^^,^^5^^]^. Regarding the high rate of nonadherence and side effects caused by an inappropriate dose of lithium treatment, the determination of the right dose of this drug is considered a significant treatment challenge^[^^6^^]^. Long-term studies support the effect of genetic variations in the various responses of individuals to medications^[^^7^^]^. Understanding the molecular pathway of the function of medications would help to find variants that alter the therapeutic response. 

One of the significant actions of lithium is investigated in tauopathies^[^^8^^]^. Evidence has also shown that lithium is prescribed as a neuroprotective agent in some neurodegenerative disorders such as Parkinson’s or Alzheimer’s diseases^[^^9^^]^. Due to the absence of gliosis (neurodegeneration marker), bipolar disorder is not considered as a classic neurodegenerative disorder, but has specific patterns of glial loss and elevated choline levels^[^^8^^]^, and is considered as a neurodevelopmental disorder^[^^10^^]^. Many studies have suggested that lithium may target agents affecting neurotransmission and signal transduction, which results in cell-surface receptor modulation and the subsequent effect on the activity of major regulatory systems. These events may result in activation of relevant transcription factors and their target genes’ expression^[^^11^^]^. Serine-threonine protein kinase-related cascades is among the main influenced signaling pathways. These cascades are reported to be activated by lithium^[^^12^^]^. Serine-threonine protein kinases are involved in the establishment of neuronal polarity, neurite outgrowth, and regulation of microtubule dynamics, axogenesis, neuronal migration, and inhibition of microtubule-dependent accumulation^[^^13^^,^^14^^]^. Among them, MARK family of protein kinases has been under focus as exerts important roles in phosphorylation of certain sites in tau protein. This phosphorylation is found to be increased in abnormal tau of Alzheimer’s brain tissue, resulting in detachment of tau and microtubules^[^^14^^]^. 

Several studies have reported similar patho-physiological processes in Alzheimer's disease and bipolar disorder. Further, impairments of episodic memory and executive dysfunction are shared between these disorders^[^^15^^-^^17^^]^. Therefore, it is expected that these two disorders represent some common genetic basis and overlap in some pathogenic signaling pathways. The *MARK2* gene has been reported to be associated with Alzheimer's disease and bipolar disorder. This gene is widely expressed in the brain throughout the life and has shown spatial and temporal relationships with both neurodevelopmental and neurodegenerative processes^[^^10^^]^. Activated MARK2, along with neurofibrillary tangles, is located in the same area in the brains of Alzheimer’s patients. Also, more target areas of MARK2 on tau in transgenic mice of the Tauopathies model emphasize the importance of MARK2 in this disease^[^^18^^]^.

Lithium is prescribed in Alzheimer's disease to modulate tau phosphorylation and amyloid-β production, as well as improve memory impairment^[^^19^^]^. Lithium has been shown to reduce GSK3-β activity^[^^20^^]^. It has been demonstrated that GSK3-β is responsible for MARK2 phosphorylation and activation, which give rise to tau phosphorylation at Ser-262^[^^21^^,^^22^^]^. Tau phosphorylation at this particular site is mainly mediated by a GSK-3β MARK2 pathway^[^^23^^]^. Hence, common variants of *MARK2* gene could be investigated as the candidate modulators of lithium therapeutic action in Alzheimer’s disease and bipolar disorder. 

One diverse study has identified *MARK2* as an upstream regulator of PINK1, which raises a new perspective on the mitochondrial transport status of neurons^[^^24^^]^. The motor proteins dynein (retrograde) and kinesin (anterograde) are responsible for mitochondria transport within a normal neuron. A molecular switch, PINK1, modulates the ability to carry mitochondria between dynein and kinesin. MARK2 regulates the direction of neuronal mitochondrial movement by cleavage and binding/phosphorylation of PINK1^[^^25^^,^^26^^]^. Apart from the important role of MARK2 protein in the brain, especially in the pathophysiology of neuro-developmental disorders, due to its serine-threonine kinase activity, MARK2 protein may also play a role in the lithium metabolic pathway^[^^12^^]^. It has been established that lithium activates the serine-threonine kinase Akt-1^[^^27^^]^. Overexpression of MARK2 has been shown to provoke PI3K/AKT/NFκB signaling pathways and induces cell apoptosis^[^^28^^]^. This evidence also supports the role of *MARK2* variants in the lithium’s mechanism of action and the patients’ various responses to the medications. Regarding the importance of *MARK2* gene in the pathophysiology of bipolar disorder and also in the lithium’s mechanism of action, the association of one of these gene variants, rs10792421, was investigated with different responses to lithium treatment in bipolar type 1 patients.

## MATERIALS AND METHODS

**Study subjects**

The sample size was assessed using the usual expectations of this type of study, which is 95% CI (α = 0.05), 80% power (β = 20%), and minimum extreme odds ratio to detect 2.0. According to data from the Iranome database (www.iranome.com), the hypothetical percentage of controls with exposure to risk alleles was set at 0.28. All patients were diagnosed by psychiatrists at Imam Hossein Hospital of Tehran and Razi Hospital of Urmia according to the Mental Illness Diagnosis and Statistics Manual, 4^th^ Edition Text Revision (DSM-IV-TR) and DSM-5^[^^29^^]^. Due to the naturalistic design of the study, patients receiving other pharmacological treatments and had stable doses of other treatments for at least four weeks prior to the start of lithium were included in the study. Various patients’ responses to lithium carbonate drugs were calculated using the Clinical Global Impression Severity (CGI-S) scale. Although the CGIS is a common clinical scale for psychiatric disorders, it relies heavily on the clinical understanding of a patient's mental state by a psychiatrist and includes two subtypes. The first subclass ranges from 1 (no illness) to 7 (very serious illness) and the second subclass measures global improvement of patients from the beginning of their disorder. The severity of the illness was assessed by a psychiatrist through diagnosing the current state of the illness at the start of treatment. Patients were eventually divided into three groups: responders (R; n = 101), semi-responders (SR; n = 91), and non-responders (NR; n = 11). The information collected from all cases includes social demographic information (age, gender, ethnicity, marriage history, vocational status, and education); lithium carbonate consumption data (amount, side effects, and age of start); codependence: all other drugs, including alcohol and tobacco; current treatment and medication; medical history, including height and weight for calculating BMI; family history and drug-related problems. All patients were treated with lithium and followed for at least six months. Information on dose and drug consumption behavior, including results of blood lithium levels, routinely collected in the clinic as part of routine clinical care, was also collected.

**Genotyping methods**

Peripheral blood (4 ml) was collected from all patients in EDTA tubes. Salting out method was employed to extract DNA from samples. 4P-ARMS-PCR method was used for genotyping the *MARK2* gene, rs10792421, in Flex Thermo Cycler (Analytik Jena, Germany). Each amplification reaction contained 2× Master Mix Red (Ampliqon, Denmark), 5 pmol/l of inner primers, 10 pmol/l of outer primers, 150 ng of DNA, and 12.5 μl of Taq DNA Polymerase. The PCR program included 35 cycles of initial denaturation at 95 °C for 5 minutes, 45 seconds at 95 °C (denaturation), 45 seconds at 55 °C (annealing), 45 seconds at 72 °C (elongation), and final extension at 72 °C for 5 minutes. The rs10792421 allele amplification produced 210, 280, and 441 bp bands for the A allele, G allele, and external primers, respectively. PCR products were further approved by random sequencing of about 15% of the genotypes using the ABI 3730 XL DNA Analyzer (Macrogen, South Korea).

**Statistical analysis**

The chi-square test was used to compare the genotypes of the patients to determine their suitability for the Hardy-Weinberg equilibrium. The association between allele and genotype frequency and response to lithium therapy was investigated using the chi-square test in three different genetic models, including recessive, dominant, and co-dominant. The results of the association analysis are shown as odds ratios and their 95% CI. *p* value less than 0.05 was considered as statistically significant in all analyses.

## RESULTS

The association between rs10792421 and patients’ responses to lithium was investigated in 203 bipolar I patients through Pearson’s chi-squared analysis. The obtained results proved a significant association of rs10792421 different genotypes with patients’ responses to lithium in bipolar I patients. In R group, as shown in Table 1, the A allele was more frequent compared to NR group in codominant model of analysis (OR: 11.40; 95% CI: 1.28–101.37; *p* = 0.028). AA and AG genotypes were more frequent in R group compared to NR group in the dominant model of analysis (OR: 4.86; 95% CI: 1.35–17.55; *p* = 0.017). Based on Table 2, in SR, the A allele was more frequent in R group when compared with NR group in codominant model of analysis (OR: 9.33; 95% CI: 1.04–84.09; *p* = 0.042). AA and AG genotypes were more frequent in the SR group compared NR group in the dominant model of analysis (OR: 4.87; 95% CI: 1.33–17.75; *p* = 0.018).

## DISCUSSION

MARK2 kinase is recruited in a variety of neuronal processes such as polarity, motility, neuronal migration, and also tau protein phosphorylation^[^^25^^,^^30^^]^, which is essential for cortical formation^[^^31^^]^. The immature neurons’ migration is considered as an essential step for cortical formation^ [^^31^^]^. Bipolar disorder has been identified as a neurodevelopmental disorder based on obtained results of cortical cell migration incompetence in patients^[^^32^^,^^33^^]^. Abnormal neuronal migration can also be observed later in the life of patients with Alzheimer's disease^[^^17^^]^. Tauopathy is recognized as one of the pathophysiological symptoms of Alzheimer's disease^[^^34^^]^. In a 2013 study, Gu *et al.*^[^^13^^]^ found that MARK2 enhances tau phosphorylation *in vitro * and reported certain interactions between MARK2 and tau in the brain tissue of postmortem patients with Alzheimer's disease. The role of tauopathy in bipolar disorder has also been investigated. A research on cerebrospinal fluid in youth bipolar patients and a similar study in elderly patients and mild cognitive disorders detected no proof of of tauopathy^[^^35^^,^^36^^]^. However, in another study, it has been demonstrated that the overall proportion of tau-phosphorylated reduced in bipolar patients who carry the high-risk allele variant of the previously studied *CACNA1C* gene^[^^35^^]^. This reduction was not observed in healthy individuals carrying the same allele. Further studies are needed to study the association of tau phosphorylation by regulatory genes, such as the *MARK2* gene, with bipolar disease to confirm previous findings and attribute them to bipolar disorder^[^^36^^]^.

**Table 1:** Association analysis of rs10792421 with response to lithium carbonate in responder group (n=112)

**Model of inheritance**	**Genotypes**	**NR number (%)**	**R number ** **(%) **	**OR ** **(95% CI)**	***p*** **value**
Codominant	G/G A/G A/A	6 (54.4) 4 (36.4) 1 (9.1)	20 (19.8) 43 (42.2) 38 (37.6)	1.00 3.23 (0.82-12.72) 11.40 (1.28-101.37)	0.028
					
Dominant	G/G A/G-A/A	6 (54.5) 5 (45.5)	20 (19.8) 81 (80.2)	1.00 4.86 (1.35-17.55)	0.017
					
Recessive	G/G-A/G A/A	10 (90.9) 1 (9.1)	63 (62.4) 38 (37.6)	1.00 6.03 (0.74-49.00)	0.038
					
Overdominant	G/G-A/A A/G	7 (63.6) 4 (36.4)	58 (57.4) 43 (42.6)	1.00 1.30 (0.36-4.71)	0.69
					
Log-additive	---	---	---	3.32 (1.28-8.62)	0.0074

*NR, non-responders; R, responders*

Lithium has various molecular targets, including GSK3-β inhibitor^[^^37^^]^. There is conflicting evidence that GSK3-β ultimately inhibits or activates MARK2^[^^21^^,^^38^^]^. As a result, it is not yet clear whether lithium therapy can reduce or increase tau phosphorylation in carriers of the common variant of the *MARK2* gene^[^^10^^]^. Lithium has been found to regulate apoptosis through NF-kappa B and mitogen-activated protein kinase-dependent pathways^[^^39^^]^. NF-kappa B is an important mediator and an essential transcription factor in inflammatory signaling and is involved in the relapse/remission course of bipolar disorder^[^^40^^,^^41^^]^. One of the *MARK2* gene variants, rs10792421, has been indicated to alter the NF-kappa B motif^[^^42^^]^. It has also been reported that this variant may act in a similar way in both Alzheimer's disease and bipolar disorder. These results make this variant a potential marker in the pathophysiology of bipolar disorder and lithium’s mechanism of action.

The current study investigated the association of rs10792421 of *MARK2* gene with the response to lithium medication in bipolar type 1 patients. Analyses showed that the AA genotype of this variant is associated with better response to lithium treatment in bipolar type 1 patients, as in comparative studies, AA genotype was more frequent than GG and AG + GG genotypes in the responder to lithium group, and also in the moderate responder to lithium group than the non-responder group. Therefore, the A allele of this variant can serve as a low-risk allele for treatment with the standard dose of lithium in bipolar patients.

Overall, it could be concluded that patients with AA genotype may benefit more from the standard dose of lithium than those with GG or AG genotypes. 

**Table 2:** *Association analysis of rs10792421 with response to lithium carbonate in semi* *-* *responders*

**Model of inheritance**	**Genotypes**	**NR number** ** (%)**	**R number** ** (%)**	**OR** ** (95% CI)**	***p*** **value**
Codominant	G/G A/G A/A	6 (54.4) 4 (36.4) 1 (9.1)	18 (19.8) 45 (49.5) 28 (30.8)	1.00 3.75 (0.95-14.88) 9.33 (1.04-17.75)	0.042
					
Dominant	G/G A/G-A/A	6 (54.5) 5 (45.5)	18 (19.8) 73 (80.2)	1.00 4.87 (1.33-17.75)	0.018
					
Recessive	G/G-A/G A/A	10 (90.9) 1 (9.1)	63 (69.2) 28 (30.8)	1.00 4.44 (0.54-36.41)	0.098
					
Overdominant	G/G-A/A A/G	7 (63.6) 4 (36.4)	46 (50.5) 45 (49.5)	1.00 1.71 (0.47-6.25)	0.41
					
Log-additive	---	---	---	3.30 (1.21-9.02)	0.012

NR, non-responders; R, responders

Moreover, patients with GG genotype of rs1079241 have more challenges with the routine treatment of lithium and are required to be carefully monitored during their treatment to avoid harmful side effects, suicide, or relapse of disorder.

## DECLARATIONS

### Ethical statement

The above-mentioned sampling protocols were approved by the Institutional Review Board of Shahid Beheshti Medical University, Tehran, Iran (ethical code: IR.SBMU.MSP.REC.1398.344). All participants signed consents in accordance with the Declaration of the World Medical Association in Helsinki. 

### Data availability

The analyzed data sets generated during the study are available from the corresponding author on reasonable request.

### Author contributions

SSAA and MDO made substantial contributions to the conception of the study and supervised the study. AA performed the laboratory assessment. JS, SH, and BB collected samples and analyzed the data. AO wrote the manuscript. All authors have approved the final manuscript and have agreed both to be personally accountable for their own contributions and to ensure that questions related to the accuracy or integrity of any part of the work are appropriately investigated, resolved, and the resolution documented in the literature.

### Conflict of interest

None declared.

### Funding/support

This study was financially supported by Shahid Beheshti University of Medical Sciences, Tehran, Iran.
